# Minimal invasive management of traumatic transection of the vertebral artery

**DOI:** 10.1186/2193-1801-3-206

**Published:** 2014-04-28

**Authors:** Steve MM de Castro, Sarah C Christiaans, Rene van den Berg, Niels WL Schep

**Affiliations:** Department of Traumatology, Academic Medical Center, Meibergdreef 9, 1105 AZ Amsterdam, The Netherlands; Department of Radiology, Academic Medical Center, Meibergdreef 9, 1105 AZ Amsterdam, The Netherlands

## Abstract

Stab wounds to the neck can be potentially lethal. They are often associated with vascular injury of the carotid artery and jugular vein. Injury of the vertebral artery is rarely seen. The injury can vary from severe bleeding after transection with hemorrhage into the surrounding soft tissues of the neck to dissection and separation of the intimal lining from the subjacent media of an artery and subsequent occlusion of the vessel. We report a case of traumatic vertebral artery transection managed by minimal invasive balloon occlusion.

## Introduction

Injury to the cervical carotid arteries are a well-recognized phenomenon in sharp and even blunt neck trauma. Injury to the vertebral artery is less common. The deep location of the vertebral artery within the neck makes injury relatively less common. This deep location also makes it difficult to detect and manage (Atar et al. 2005). The widespread availability of computed tomography angiography (CTA) has aided in the prompt detection of these injuries. The incidence of cervicocranial arterial injuries secondary to penetrating trauma varies from 1–7%. (Simionato et al. 1999; Kobernick & Carmody 1984; Cohen et al. 2005). We report a case of traumatic sharp transection of the extracerebral vertebral artery managed by minimal invasive balloon occlusion.

Our objectives for reporting this case are to show an extremely rare case of traumatic transection of the vertebral artery with an actively hemorrhaging vessel and to discuss the treatment.

## Case presentation

Consent was obtained from the patient to publish his case, and the study was authorized by the local ethical committee and performed in accordance with the ethical standards of the 1964 Declaration of Helsinki as revised in 2000.

The institutional review board and Medical Ethics Committee at the Academic Medical Center approved the case-report as it is.

**Figure 1:**
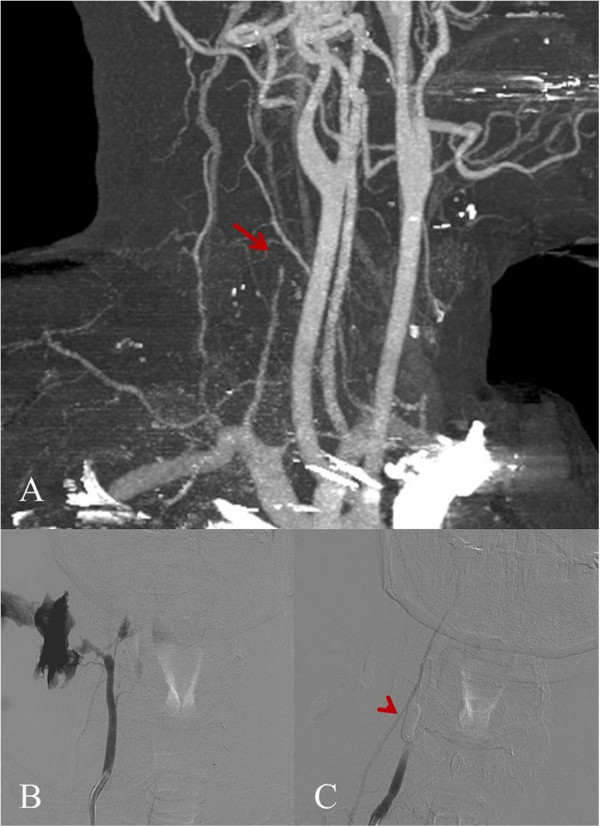
**Anterolateral view of CTA (A) and frontal projection of right vertebral artery on conventional angiogram (B and C). A**. Tapering of the right vertebral artery (VA) is shows with the arrow. **B**. The bleeding, which started during navigation to the right VA. **C**. The stable situation after inflation of the detachable balloon is shown with the arrowhead.

A 39-year-old male with no history of past illness presented to our emergency department after receiving multiple stab wounds to the hands and neck area. Ambulance paramedics reported profuse bleeding from the neck which stopped in the ambulance after putting pressure over the wound. Primary survey revealed an agitated patient with normal breath sounds. Vital signs showed a SpO_2_ of 98%, a tachycardia of 130/minute with a blood pressure of 100/80 mmHg. Complete physical examination of the patient revealed an anteromedial stab wound to the neck with no signs of active bleeding and multiple lacerations to the hands. A CTA of the neck and brain showed tapering of the right vertebral artery (VA) ending in a complete stop (Figure 1A) due to a traumatic transection. The patient was scheduled to undergo emergency angiography with preventive occlusion of the right VA. First the normal left (contralateral) VA was catheterized to check the patency of the vertebrobasilar circulation. Just at the time of navigation to the right VA, the patient developed a severe rebleed (Figure 1B), which was controlled by local compression over the wound by the surgical assistant. After exchange for a 6 French guiding catheter, a detachable balloon was inflated over the transection, resulting in local and hemodynamic stabilization (Figure 1C). A control angiogram was performed through both the left VA as well as the occipital artery and showed no retrograde filling of the transection. A control CTA was performed 7 days later and showed a stable occlusion of the right VA both antegrade and retrograde from the transection. The patient made an uneventful recovery and was discharged from the hospital on day seven.

## Discussion

Traumatic VA bleeding is difficult to diagnose and manage because of the deep location of the injured vessel. Surgical access is much less straightforward than carotid artery laceration, and therefore an endovascular approach is becoming first choice in management. (Demetriades et al. 1996) Several authors have successfully attempted an endovascular approach before. (Herrera et al. 2008; Lee et al. 2007; Nutting et al. 2003).

Cohen et al. (Cohen et al. 2005) successfully embolized the proximal and distal end of a hemorrhaging VA using coils. Other cases have been published, but are few in number. (Atar et al. 2005; Kobernick & Carmody 1984; Chaara et al. 2001; Hetz et al. 1989; Ben-Menachem et al. 1987; Herrera et al. 2008; Lee et al. 2007; Nutting et al. 2003) Alternative methods to coil embolization include various other embolic materials. Simionato et al. (Simionato et al. 1999) successfully used histoacryl and lipiodol to treat a patient with a VA bleeding secondary to a closed-head motorcycle accident. Detachable balloon occlusion as described in this case-report is also an alternative but has never been described before to our knowledge.

Unfortunately, these endovascular techniques and most surgical interventions result in sacrifice of the injured VA. Recent development of covered stents could result in salvage of the injured vessel. (Cox et al. 2007; Schonholz et al. 2007). This can only be achieved if the injured vessel can be traversed with a guiding wire.

Surgical repair of a VA transection is indicated if the vertebrobasilar circulation is not patent. If this patency is not restored, it will result in posterior circulation ischemia and severe neurological symptoms. In some cases, temporary endovascular occlusion may be useful to stabilize the patients and organize a definite surgical intervention. Repair consists of surgical exploration at the site of injury. The injured vessel can then be trapped and ligated and subsequent revascularization can be performed. This can be achieved by anastomosing the VA to the external carotid artery. If this is not possible, various types of auto- and allografts can be used to make a bypass between VA and external carotid artery.

The advantage of an endovascular approach over a surgical approach is the shorter time frame and comparative simplicity in which bleeding may be controlled. The management is influenced by several factors including the complexity and location of the transection and the hemodynamic stability of the patients. Other factors, including the availability of the surgical and radiological teams also play a substantial role.

Patients with VA transection should undergo emergency angiography and embolization (or balloon occlusion) if the vertebrobasilar circulation permits this. If the vertebrobasilar circulation is at risk, a covered stent can be placed if the transection can be traversed. If this is unsuccessful a temporary occlusion should be performed to control the bleeding and subsequent definite repair should be performed.
